# Brain and CSF Alzheimer’s Biomarkers Are Associated with SERPINE1 Gene Expression

**DOI:** 10.3390/genes16070818

**Published:** 2025-07-12

**Authors:** Cynthia Picard, Henrik Zetterberg, Kaj Blennow, Sylvia Villeneuve, Judes Poirier, on behalf of the PREVENT-AD Research Group

**Affiliations:** 1 Douglas Mental Health University Institute, Montréal, QC H4H 1R3, Canada; cynthia.picard@mail.mcgill.ca (C.P.); sylvia.villeneuve@mcgill.ca (S.V.); 2 Centre for the Studies on Prevention of Alzheimer’s Disease, Montréal, QC H4H 1R3, Canada; 3 Department of Psychiatry and Neurochemistry, Institute of Neuroscience and Physiology, The Sahlgrenska Academy, University of Gothenburg, SE-405 30 Gothenburg, Sweden; henrik.zetterberg@clinchem.gu.se (H.Z.); kaj.blennow@neuro.gu.se (K.B.); 4 Clinical Neurochemistry Laboratory, Sahlgrenska University Hospital, 431 30 Mölndal, Sweden; 5 Department of Neurodegenerative Disease, UCL Institute of Neurology, London WC1N 3BG, UK; 6 UK Dementia Research Institute at UCL, London WC1E 6BT, UK; 7 Hong Kong Center for Neurodegenerative Diseases, Hong Kong 1501-02 15, China; 8 Wisconsin Alzheimer’s Disease Research Center, University of Wisconsin School of Medicine and Public Health, University of Wisconsin-Madison, Madison, WI 53715, USA; 9 Centre for Brain Research, Indian Institute of Science, Bangladore 560012, India; 10 Department of Psychiatry, McGill University, Montréal, QC H3A 0C8, Canada

**Keywords:** SERPINE1, Alzheimer’s disease, biomarkers

## Abstract

Background: SERPINE1, also known as plasminogen activator inhibitor (PAI), has been proposed as a potential blood biomarker for the early detection and diagnosis of Alzheimer’s disease (AD). Expanding on previous studies, this research contrasted SERPINE1 levels in CSF and brain tissue of AD patients and those at risk for AD with established AD biomarkers. Methods: Utilizing OLINK and immunoassay methods, CSF SERPINE1 protein levels were quantified across two separate cohorts: PREVENT-AD and ADNI. Microarray and RNAseq were used to measure tissue *SERPINE1* mRNA levels in two separate cohorts: the Douglas-Bell Canada Brain Bank and the Mayo Clinic Brain Bank. Results: At the pre-clinical stage, elevated CSF levels of pTau, tTau and synaptic markers, alongside reduced hippocampal volume, correlate with CSF SERPINE1 levels. Elevated cortical *SERPINE1* mRNA levels in autopsy-confirmed AD show weak correlation with regional plaques and tangles densities, but strong correlation with Braak staging. Conclusions: CSF SERPINE1 levels can be used as an early biomarker for the detection of pathological changes associated with AD. Higher SERPINE1 levels correlate more strongly with tau pathology than with amyloid formation or deposition.

## 1. Introduction

*SERPINE1* (plasminogen activator inhibitor, PAI) encodes a member of the serine proteinase inhibitor superfamily. It is one of the most important inhibitors of fibrinolytic activity thus leading to pathological fibrin deposition and tissue damage [1]. This role is attributed to the fact that SERPINE1 is the principal inhibitor of tissue plasminogen activator (tPA, encoded by the gene *PLAT*) and urokinase-type plasminogen activator (uPA, encoded by the gene *PLAU*).

Plasminogen is expressed primarily by the liver and is therefore present in the plasma, but mRNA levels have been detected in various tissues including brain, adrenal, kidney, testis, heart, lung, uterus, spleen thymus and gut [2]. The plasminogen activator system is pivotal in various physiological and pathological processes, encompassing coagulation, fibrinolysis, inflammation, wound healing and malignancy [1].

SERPINE1 is also known to inhibit cell migration by competing for vitronectin binding to integrins [3]. Recent findings reveal vitronectin plays multiple roles within the nervous system, where it contributes to activities such as neural differentiation and neurogenesis, while also having a role in regulating axon size and providing support and guidance for neurite extension [4]. By interacting with integrin receptors in vascular endothelial cells, vitronectin can reduce the permeability of the blood–brain barrier [4].

Amyloid beta (Aβ), one of the hallmarks of Alzheimer’s disease (AD), is known to increase tPA and uPA [5]. In turn, tPA and uPA cleave plasminogen (encoded by the gene *PLG*), resulting in the activation of the serine protease plasmin [5]. Plasmin was shown to degrade Aβ fibrils, therefore attenuating Aβ neurotoxicity [5]. In AD, brain tissues contain reduced levels of plasmin when compared to controls [6].

SERPINE1 was shown to accumulate in blood samples from mild cognitively impaired (MCI) and AD patients [7]. Given the established correlation between plasma SERPINE1 and cognitive impairment, SERPINE1 is proposed as a potential biomarker for early AD detection and diagnosis [7]. Consistent with this finding, *SERPINE1* hippocampal RNA prevalence is significantly increased in AD patients relative to controls [8].

In the present study, we examined SERPINE1 concentrations in control and AD brain tissue and contrasted *SERPINE1* mRNA levels as a function of neurofibrillary tangles density, senile plaque density and Braak stages. To validate SERPINE1 as a potential presymptomatic AD biomarker, we analyzed data from the PREVENT-AD cohort, which consists of asymptomatic individuals with a family history of late-onset AD. CSF SERPINE1 protein levels were measured and contrasted with classical AD pathological biomarkers such as tTau, pTau181 tau and Aβ42. We replicated our biochemical findings in living ADNI subjects spanning various disease stages.

## 2. Materials and Methods

### 2.1. Study Participants from the Douglas-Bell Canada Brain Bank Cohort

Fifty-five autopsy-confirmed AD brains and thirty autopsy-confirmed control brains were obtained from the Douglas-Bell Canada Brain Bank (see Table 1 for demographics). Based on reviews of medical records, neuropsychological assessments and care caregiver interviews, there was no indication of memory impairment, neurological or neuropsychiatric diseases in the older adults control group. Controls only exhibited neuropathology that is consistent with normal ageing, characterized by plaque and tangle densities of less than 10/mm^3^ and 20/mm^3^, respectively, in at least one section of the hippocampus and neocortex. AD cases had to fulfill the histopathological NINCDS-ADRDA criteria for definite AD [9]. Written informed consent was obtained from each participant and their study partner. All procedures were approved by the McGill University Faculty of Medicine Institutional Review Board and by the Ethics Board of the Douglas Mental Health University Institute. It complies with ethical principles of the Declaration of Helsinki and conformed to the Code of Ethics of the World Medical Association as per protocol approval number #IUSMD-02-34, 17 September 2024.

Total RNA was extracted from 100 mg of cortical brain tissues and quantified using a NanoDrop spectrophotometer ND-100 (NanoDrop Technologies, Inc, Wilmington, NC, USA). RNA integrity was assessed using a 2100 Bioanalyzer (Agilent Technologies, Saint-Laurent, QC, Canada) and samples with RNA integrity number below 3.5 were removed. Sense-strand cDNA was synthesized from 10 ng of total RNA, followed by fragmentation and labelling using the GeneChip WT Pico terminal labelling kit, in accordance with the manufacturer’s protocol (Thermo Fisher Scientific, Saint-Laurent, QC, Canada). Subsequently, 5 mg of the labelled DNA target was hybridized on GeneChip Clariom^TM^ D human (Thermo Fisher Scientific, Saint-Laurent, QC, Canada) and incubated at 45 °C in the GeneChip Hybridization oven 640 (Affymetrix, Santa Clara, CA, USA) for 17 h at 60 rpm. Post-hybridization, the arrays were washed in a GeneChips Fluidics Station 450 (Thermo Fisher, Saint-Laurent, QC, Canada) using GeneChip Hybridization Wash and Stain kit according to manufacturer’s guidelines (Thermo Fisher, Saint-Laurent, QC, Canada). Finally, the microarrays were scanned on a GeneChip scanner 3000 (Thermo Fisher, Saint-Laurent, QC, Canada). Gene expressions were normalized using the Transcriptome Analysis Software from Thermo Fisher Scientific (Saint-Laurent, QC, Canada). *SERPINE1* mRNA levels are presented on a log_2_ scale.

### 2.2. Study Participants from the Mayo Clinic/Banner Brain Bank Cohort

A total of 193 subjects from the Mayo Clinic Brain Bank and Banner Sun Health institute were included, comprising individuals with Alzheimer’s disease (AD; *n* = 82), progressive supranuclear palsy (PSP; *n* = 83) and cognitively unimpaired (CU; *n* = 28). Control subjects met the following criteria: Braak NFT stage of 3.0 or less, CERAD neuritic and cortical plaque densities of 0 (none) or 1 (sparse) and an absence of any pathological diagnoses, including AD, Parkinson’s disease, Lewy body disease, vascular dementia, progressive supranuclear palsy, motor neuron disease, corticobasal degeneration, Pick’s disease, Huntington’s disease, frontotemporal lobar degeneration, hippocampal sclerosis or dementia without distinctive histology. All participants were North American Caucasians who were aged 60 or older at the time of their death (refer to Table 1 for demographic details). All procedures complied with the ethical principles of the Declaration of Helsinki. Gene expression data were retrieved from AMP-AD Knowledge Portal https://adknowledgeportal.synapse.org (synapse ID: syn5550404, accessed on 1 November 2024). Expression profiles were generated using next-generation RNA-sequencing (RNAseq) from temporal cortex RNA samples collected for 193 individuals enrolled through the Mayo Clinic Brain Bank and Banner Sun Health research institute [10].

### 2.3. Study Participants from the Asymptomatic PREVENT-AD Cohort

The PRe-symptomatic EValuation of Experimental or Novel Treatments for Alzheimer’s Disease (PREVENT-AD) cohort consists of asymptomatic, “at-risk” individuals with a parental or multi-sibling history of sporadic AD [11]. Most participants were over the age of 60 at the time of recruitment (see Table 1 for demographics); however, individuals aged 55–59 years were eligible if they were within 15 years of the age of symptom onset of their youngest-affected relative. Cognitive normality was confirmed at the eligibility visit using the Clinical Dementia Rating (CDR) and the Montreal Cognitive Assessment (MoCA). PREVENT-AD participants were followed longitudinally, with annual visits involving cognitive evaluations, neurosensory testing, blood and CSF collection (for a subset of individuals), structural and functional magnetic resonance imaging (MRI) and positron emission tomography (PET) scans. Each participant and their study partner provided written informed consent. All study procedures were approved by the McGill University Faculty of Medicine Institutional Review Board and adhered to the ethical standards outlined in the Declaration of Helsinki. A detailed description of the PREVENT-AD cohort is available in previously published work [11].

#### 2.3.1. Cerebrospinal Fluid Measurements of the PREVENT-AD Cohort

Lumbar punctures (LP) were performed in PREVENT-AD participants using a Sprotte 24-gauge atraumatic needle following an overnight fast. To remove cells and insoluble material, CSF samples were centrifuged (~2000× *g*) for 10 min at room temperature within 4 h of collection. The resulting supernatant was aliquoted into 0.5 mL polypropylene cryotubes and stored at −80 °C.

CSF levels of SERPINE1 were quantified in a subset of PREVENT-AD participants (*n* = 125) using the OLINK Cardiovascular III panel, which utilizes proximity extension assay (PEA) technology as per manufacturer’s instructions (Uppsala, Sweden). Results are reported in arbitrary normalized protein expression units (NPX).

In the same subset (*n* = 125), CSF concentrations of AD biomarkers Aβ42, phosphorylated tau (pTau181) and total tau (tTau) were measured using the validated Innotest enzyme-linked immunosorbent assay (ELISA) kit (Fujirebio, Ghent, Belgium) following the standardized protocols established by the BIOMARKAPD consortium (Aβ42 Cat.# 81583, pTau181 Cat.# 81581 and tTau Cat.# 81579).

CSF GAP43, SNAP25 and SYT1 were immunoprecipitated and their concentrations were measured by mass spectrometry, as previously described [12]. Mass spectrometry results are expressed in arbitrary units.

#### 2.3.2. Hippocampus Volume of the PREVENT-AD Cohort

MRI acquisition protocols have been previously described in detail [13]. In brief, T_1_-weighted images for each participant were segmented into gray matter, white matter and CSF using Statistical Parametric Mapping 12 v.6225. running on MATLAB version 2012a [14]. Total intracranial volume was computed as the sum of the three segmentation volumes. Subject-specific templates were generated using the DARTEL toolbox [15] and all scans were non-linearly normalized to these templates. The resulting templates were then registered to the MNI-ICBM152 standard space using non-linear transformations with modulation for both linear and non-linear deformations. These transformation parameters were applied to each subject’s T_1_ image to align it with the template space. Following segmentation and normalization, all images underwent visual quality control. Gray matter probability (c1) maps in template space were smoothed with an 8 mm^3^ isotropic Gaussian kernel to produce grey matter volume images. These were then masked using a maximum probability grey matter mask derived from the group-average image. For comparative analyses, hippocampal volume measures were extracted in native space for all participants using a previously validated method [16].

#### 2.3.3. APOE Genotyping

The QIAsymphony apparatus and DNA Blood Mini QIA Kit (Qiagen, Valencia, CA, USA) were used to isolate DNA from brains or whole blood. The standard QIASymphony protocol was used in accordance with the manufacturer’s guidelines. The PyroMark Q96 pyrosequencer (Qiagen, Toronto, ON, Canada) was used to determine *APOE* genotype, with the following primers purchased from Eurofins genomics (Toronto, ON, Canada): rs429358 amplification forward 5’-ACGGCTGTCCAAGGAGCTG-3’, rs429358 amplification reverse biotinylated 5’-CACCTCGCCGCGGTACTG-3’, rs429358 sequencing 5’CGGACATGGAGGACG-3’, rs7412 amplification forward 5’-CTCCGCGATGCCGATGAC-3’, rs7412 amplification reverse biotinylated 5’-CCCCGGCCTGGTACACTG-3’ and rs7412 sequencing 5’-CGATGACCTGCAGAAG-3’.

### 2.4. Statistical Analysis

All statistical analysis including linear and logistic regressions were performed using JMP Pro 16.

### 2.5. Data Availability

Data collected from the Douglas-Bell Canada Brain Bank are not publicly available; however, data can be provided by the corresponding author upon reasonable request. Gene expression data from the Mayo Clinic were downloaded from AMP-AD Knowledge Portal https://adknowledgeportal.synapse.org (synapse ID: syn5550404, accessed on 1 November 2024). CSF, genetic and clinical data from the ADNI cohort were downloaded from the ADNI website (http://adni.loni.usc.edu/, accessed on 1 November 2024) following registration.

The PREVENT-AD multimodal data, including subject characteristics, CSF biomarkers levels, tau and amyloid-PET SUVR, *APOE* genotypes and RBANS scores, have been summarized in the supporting data files of the PREVENT-AD data release 7, which are available from the corresponding authors upon reasonable request at https://openpreventad.loris.ca/ and following registration.

## 3. Results

### 3.1. SERPINE1 mRNA Levels Correlate with AD Pathology

**Figure 1:**
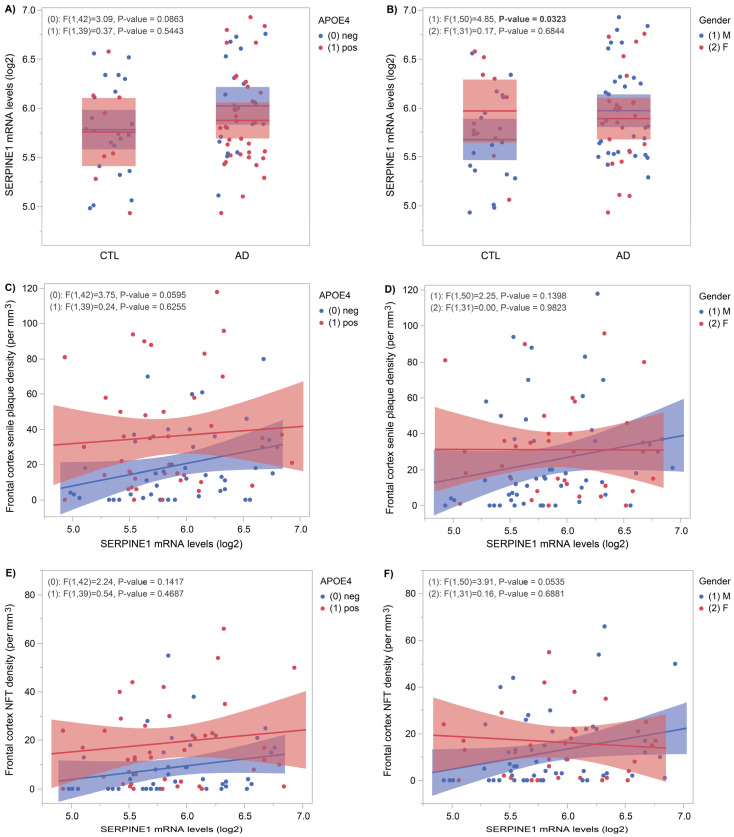
*SERPINE1* mRNA levels in control and AD frontal cortices according to *APOE4* status and gender. *SERPINE1* mRNA levels were measured using the Clariom^TM^ D microarray in 85 brain samples from controls and AD subjects from the DBCBB cohort. (**A**) in *APOE4*-negative subjects, a close to significant association was found between controls and AD subjects (*p* = 0.09). (**B**) In males only, *SERPINE1* mRNA levels were significantly higher in AD compared to controls (*p* = 0.03). (**C**) In *APOE4*-negative subjects, *SERPINE1* mRNA levels had a close to significant positive correlation with senile plaque density (*p* = 0.06). (**D**) In males or females, no significant associations were found between *SERPINE1* mRNA levels and senile plaque density. (**E**) In *APOE4*-negative or positive subjects, no significant associations were found between *SERPINE1* and NFT density. (**F**) In males only, *SERPINE1* mRNA levels had a close to significance positive correlation with NFT density (*p* = 0.05).

Figure 1 shows *SERPINE1* mRNA levels measured in frontal cortices of age-matched controls (*n* = 30) and AD *(n* = 55) subjects from the Douglas-Bell Canada Brain Bank. Stratified by *APOE4* status, Figure 1A shows a near-significant difference (*p* = 0.0863) in *APOE4*-negative subjects, whereas Figure 1B shows a significant increase in AD males versus CTL males (*p* = 0.03). When stratified by *APOE4* status (Figure 1C), a near-significant positive correlation (*p* = 0.06) emerged between *SERPINE1* mRNA levels and senile plaque density. Despite the gender stratification shown in Figure 1D, no significant correlations were found with senile plaque density. When comparing with neurofibrillary tangles density, no significant correlations appeared after stratifying by *APOE4* status (Figure 1E). After sex-specific analysis (Figure 1F), a significant correlation (*p* = 0.05) between *SERPINE1* mRNA levels and NFT density was found only in males.

**Figure 2:**
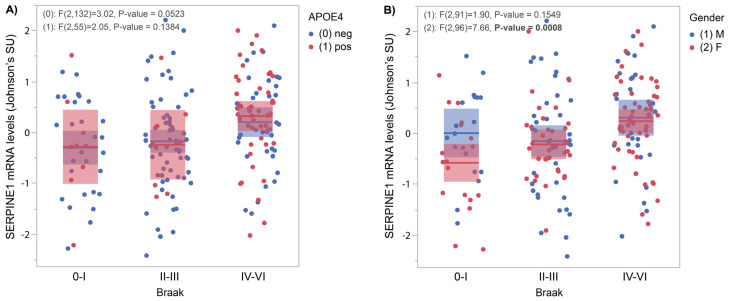
*SERPINE1* mRNA levels compared to Braak stages. *SERPINE1* mRNA levels were measured using RNAseq in 193 frontal cortices from the Mayo clinic cohort and contrasted with *APOE4* and gender. (**A**) In *APOE4*-negative subjects, correlations were found at a trend level between *SERPINE1* mRNA levels and Braak stages (*p* = 0.05). (**B**) In females only, *SERPINE1* mRNA levels increased in relation to Braak stages (*p* = 0.0008).

Figure 2 shows *SERPINE1* mRNA levels measured by RNAseq in frontal cortices obtained from the Mayo Clinic/Banner brain bank cohort. Samples include 111 non-AD subjects (28 CTLs and 83 PSP) and 82 AD subjects for which Braak stages were split into three groups. Figure 2A shows a week relationship with *APOE4* status (*p* = 0.05); Figure 2B shows a correlation between *SERPINE1* mRNA levels and Braak stages (*p* = 0.0008) only in females.

### 3.2. CSF SERPINE1 Protein Levels Correlate Positively with AD Biomarkers

**Figure 3:**
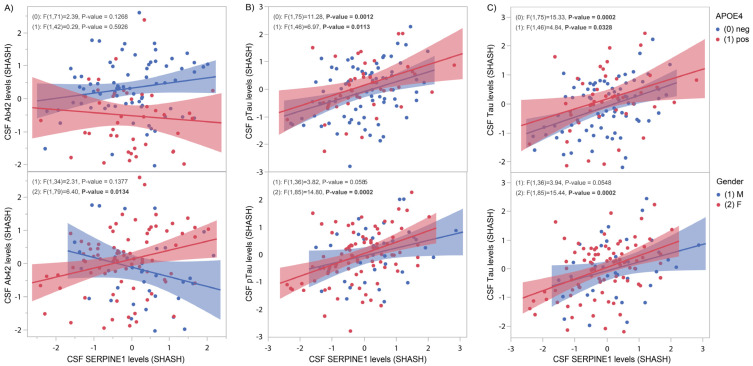
CSF SERPINE1 protein levels compared to Aβ, pTau and tTau. CSF SERPINE1 levels were measured with the help of OLINK technology in 125 CSF samples from the PREVENT-AD cohort. (**A**) In females only, SERPINE1 protein levels were significantly positively correlated with CSF Aβ (*p* = 0.01). (**B**) In *APOE4*-negative and -positive, as well as in female, subjects, a significant positive correlation was found between SERPINE1 protein levels and CSF pTau (*p* = 0.0012, *p* = 0.01 and *p* = 0.0002, respectively). (**C**) In *APOE4*-negative and -positive, as well as in female, subjects, a significant positive correlation was found between SERPINE1 protein levels and CSF tTau (*p* = 0.0002, *p* = 0.03 and *p* = 0.0002, respectively).

In a cohort of 125 “at-risk” but cognitively unaffected PREVENT-AD individuals, CSF samples were used to measure protein levels of SERPINE1 and the AD biomarkers Aβ42, pTau181 and tTau. Positive correlations with Aβ42 (Figure 3A, *p* = 0.01), pTau181 (Figure 3B, *p* = 0.0002) and tTau (Figure 3C, *p* = 0.0002) were found exclusively in females. *APOE4*-negative subjects display positive correlations with pTau181 (Figure 3B, *p* = 0.001) and tTau (Figure 3C, *p* = 0.0002). In *APOE4*-positive subjects, positive correlations were found with pTau181 (Figure 3B, *p* = 0.01) and tTau (Figure 3C, *p* = 0.03).

### 3.3. SERPINE1 Protein Levels Correlate Negatively with Hippocampus Volume

**Figure 4:**
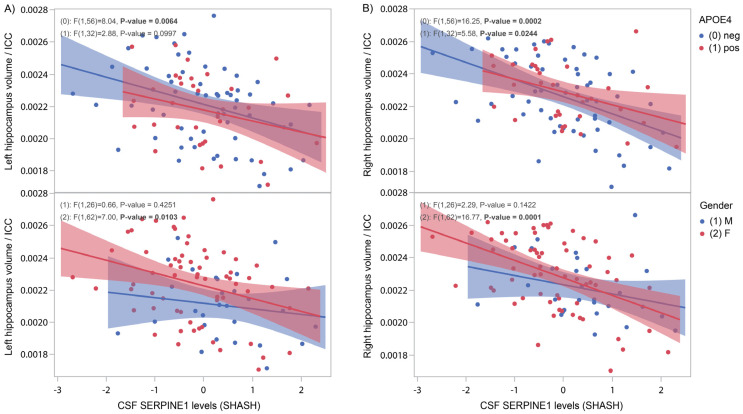
CSF SERPINE1 protein levels compared to hippocampus volume normalized against intracranial cavity. CSF SERPINE1 levels were measured with the help of OLINK technology in 125 CSF samples from the PREVENT-AD cohort. (**A**) In *APOE4*-negative subjects (**upper** panels), a significant negative correlation was found between SERPINE1 protein levels and left and right hippocampus volume (*p* = 0.006 and *p* = 0.0002, respectively). Significance was also reached between *APOE4*-positive subjects and right hippocampus volume (*p* = 0.02). (**B**) In female subjects (**lower** panels), a significant negative correlation was found between SERPINE1 protein levels and left and right hippocampus volume (*p* = 0.01 and *p* = 0.0001, respectively).

In a subset of participants from PREVENT-AD undergoing MRI (*n* = 92), hippocampus volume was measured and contrasted with CSF SERPINE1 protein levels. In *APOE4*-negative subjects, a strong negative correlation was found with both left (Figure 4A, *p* = 0.006) and right (Figure 4B, *p* = 0.0002) hippocampus volume. Analysis by gender revealed a significant inverse correlation between hippocampus volume and the outcome measure only in females (Figure 4A, left: *p* = 0.01; and Figure 4B, right: *p* < 0.0001).

### 3.4. SERPINE1 Protein Levels Correlate Positively with Synaptic Markers

**Figure 5:**
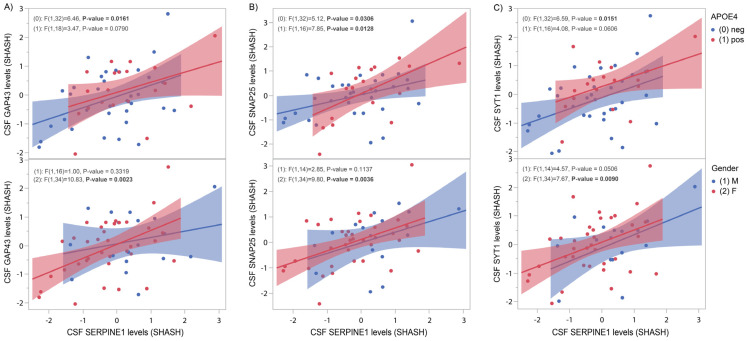
CSF SERPINE1 protein levels compared to synaptic markers GAP43, SNAP25 and SYT1. CSF SERPINE1 levels were measured with the help of OLINK technology in 125 CSF samples from the PREVENT-AD cohort. (**A**) In both *APOE4*-negative and female subjects, significant positive correlations were found between CSF SERPINE1 protein levels and GAP43 (*p* = 0.02 and *p* = 0.002, respectively). (**B**) In *APOE4*-negative and -positive and female subjects, significant positive correlations were found between CSF SERPINE1 protein levels and SNAP25 (*p* = 0.03, *p* = 0.01 and *p* = 0.004, respectively). (**C**) CSF SYT1 levels correlate with SERPINE1 at a trend level in *APOE4*-positive subjects and males (*p* = 0.06 and *p* = 0.05, respectively). Significant correlations were found between SYT1 and SERPINE1 levels in *APOE4*-negative subjects and in females (*p* = 0.02 and *p* = 0.009, respectively).

Figure 5 shows significant positive correlations between CSF SERPINE1 protein levels and three synaptic markers: GAP43 (Figure 5A, *APOE4* neg, *p* = 0.016), SNAP25 (Figure 5B. *APOE4* neg, *p* = 0.03; *APOE4* pos, *p* = 0.013) and SYT1 (Figure 5C, *APOE4* neg, *p* = 0.015). Figure 5 (lower panels) reveals significant gender-specific correlations between SERPINE1 and GAP43 (Figure 5A, females, *p* = 0.002), SNAP25 (Figure 5B, females, *p* = 0.0036) and SYT1 (Figure 5C, males, *p* = 0.05; females, *p* = 0.009).

### 3.5. Quantitative Trait Loci Analysis of CSF SERPINE1 Levels Identifies Naturally Occurring Polymorphisms in the SERPINE1 and VAT1L Genes

Using trans-pQTL analysis, we identified a genome-wide significant polymorphism (rs17705051 downstream of the *VAT1L* gene, Supplemental Figure S4) that affects SERPINE1 levels in the CSF of cognitively unaffected PREVENT-AD subjects. Rs6092, a coding SNP, was identified as the most significant cis-pQTL influencing polymorphism using CSF SERPINE1 protein levels in the ADNI cohort (Supplemental Figure S5). While the presence of these variants has no impact on AD risk levels (Table 2) in three cohorts (ADNI, MAYO, DBCBB), they significantly affect protein concentrations of SERPINE1 in the PREVENT-AD and ADNI cohorts and the mRNA levels in the MAYO cohort. As for AD biomarkers, only the presymptomatic PREVENT-AD cohort display significant association between the vesicle amine transport 1 like (*VAT1L*) variant and pTau181 (*p* = 0.006) as well as total tau levels in the CSF (*p* = 0.005).

## 4. Discussion

SERPINE1 was originally identified as a negative regulator of fibrinolysis by preventing the dissolution of blood clots [17]. Increased concentration of SERPINE1 in plasma can cause venous thrombosis and myocardial infarction [18]. More recently, myocardial infarction was proposed to be a predisposing factor for AD development [19]. Mounting evidence suggests that the ageing of the neurovasculature is a critical determinant of brain ageing and AD risk [20]. In neurodegenerative conditions, such as Alzheimer’s disease, Parkinson’s disease and frontotemporal lobar degeneration, plasma levels of SERPINE1 were found in higher concentrations [21,22]. These associations prompted us to examine SERPINE1 neurobiology throughout AD spectrum, i.e., before and after AD onset in multiple living and autopsied cohorts.

Using autopsy-confirmed AD brains, we showed that cortical *SERPINE1* mRNA levels are significantly higher compared to CTL brains (Figure 1B, *p* = 0.03). This association is also slightly affected by both *APOE4* status and gender. These results are consistent with the previous literature reporting the upregulation of *SERPINE1* mRNA levels in the superior frontal gyrus, entorhinal cortex and hippocampus of AD subjects compared to age-matched controls [8]. Only AD patients without *APOE4* in the ADNI CSF cohort showed increased SERPINE1 (Figure S1).

Plasmin, when inhibited by SERPINE1, is known to facilitate the degradation of Aβ fibrils [5] and is found in low concentrations in AD versus control brains [6]. In this context, it was tempting to propose that SERPINE1 could affect senile plaque density in a regional and concentration-dependent manner. In Figure 1C, we found such interaction at a trend level but only in *APOE4*-negative subjects. In the same vein, we examined the influence of *SERPINE1* on neurofibrillary tangles density and found a weak correlation between the two, but only in males (Figure 1F).

Using a replication cohort from the Mayo brain bank with a larger sample size, we investigated the influence of *SERPINE1* on tangle spreading using Braak staging [23]. A positive association was found between cortical *SERPINE1* mRNA levels and Braak stages in females only (Figure 2B). It is of note that the proportion of females was higher in the AD group compared to non-AD, which could explain, at least in part, those results. Nevertheless, a positive correlation with both NFT (Figure 1F) and Braak (Figure 2) suggests a possible influence on Tau metabolism and/or deposition.

To determine if SERPINE1 could be used as an early, presymptomatic, AD biomarker, we turned to the PREVENT-AD cohort, comprising asymptomatic “at-risk” individuals. Figure 3 shows strong correlations between CSF SERPINE1 protein levels and pTau181 and tTau, especially in females and *APOE4*-negative subjects (see also Figure S2 for ADNI results). These results are consistent with the tangles/*SERPINE1* association described earlier in fully established AD cases. Note that for Aβ, the expected negative correlation with *SERPINE1* was not established (Figure 3A and Figure S2A).

Using proteomic quantitative trait loci analysis, we identified genome-wide and locus-wide significant (naturally occurring) polymorphisms that display significant association with SERPINE1 protein levels in the CSF of our asymptomatic cohort of subjects (Supplemental Figures S4 and S5). The two variants that modulates CSF SERPINE1 concentration, rs17705051 (*VAT1L*) and rs6092 *(SERPINE1*) failed to affect AD risk levels when assessed in our three distinct cohorts (Table 2). Using the larger publicly available IGAP GWAS database (https://www.nature.com/articles/ng.2802, accessed on 1 November 2024), we performed additional screening for AD risk in this large dataset (*n* = 74,046) and found no significant genome-wide association with sporadic AD for rs17705051 (*p* = 0.1779) nor for rs6092 (*p* = 0.002).

A previous study involving the ADNI cohort identified an intronic SNP in *VAT1L* in association with cognitive decline measured by ADAS-cog [24]. In our asymptomatic cohort, the SNP affecting CSF SERPINE1 protein levels was found downstream of the *VAT1L* gene. Further studies will be necessary to establish the link between VAT1L and SERPINE1. The cis-acting SNP rs6092 is a coding SNP associated with impaired secretion of SERPINE1 and hemorrhage problem [25]. Other SNPs in *SERPINE1* were shown to affect AD risk [26,27].

In the early stages of AD, there is a swift degeneration of hippocampal tissue, which can be measured by magnetic resonance imaging [28]. In the PREVENT-AD cohort, we show that CSF SERPINE1 correlates inversely with hippocampus volume, particularly in females and *APOE4*-negative subjects (Figure 4 and Supplemental Figure S3 for ADNI results). These results suggest that in the absence of symptoms in at-risk individuals, hippocampal loss can be predicted by the presence of high levels of SERPINE1 in the CSF.

In preclinical stages of AD, changes in synaptic proteins were shown to precede CSF markers of neurodegeneration [29]. Increased concentrations of the presynaptic markers GAP43, SNAP25 and SYT1 were observed in the CSF during neurodegeneration [12,30,31]. In the asymptomatic PREVENT-AD cohort, we found that CSF SERPINE1 positively correlates with those three pre-synaptic markers in both *APOE4*-negative and -positive subjects (Figure 5, upper panels) as well as in all females (Figure 5, lower panels). Previous studies suggest that cognitive deficit is more closely associated with abnormalities in synaptic structure and function than with the quantity of plaques and tangles [21,32].

Our research indicates a connection between SERPINE1 and the advancement of AD, most notably in the disease’s asymptomatic period. More specifically, SERPINE1 correlates with early signs of neurodegeneration, namely with elevated levels of tTau and a reduction in hippocampus volume. In parallel, SERPINE1 is strongly linked to pTau181 tau alterations in the CSF and the presence of high concentration of synaptic markers. Over time, when AD pathology sets in, *SERPINE1* mRNA levels are elevated and show a weak correlation with plaques and NFT densities but a more significant correlation with tangle spreading monitored by Braak staging.

Our results support the notion that SERPINE1 can be used as an early biomarker for the detection of AD-associated pathological changes in asymptomatic subjects with a parental history of AD. Interestingly, most of the significant results are observed in *APOE4*-negative subjects, who are usually at lower risk of developing AD. SERPINE1’s strongest association is with tau pathology, a surprising finding considering the plasminogen activator system’s role in Aβ fibril degradation. SERPINE1’s biological network includes proteins such as PLAT, TP53 and IL6 that could possibly have an indirect effect on Tau phosphorylation via the main kinase GSK3B (Figure S6).

Further investigations are necessary to determine if the pharmacomodulation of SERPINE1 could be used to modulate disease progression.

## Figures and Tables

**Table 1:** Demographics of the three cohorts.

	DBCBB		Mayo		PREVENT-AD
	CTL (*n* = 30)	AD (*n* = 55)	non AD (*n* = 111)	AD (*n* = 82)	CU at risk (*n* = 125)
Age (mean ± SEM)	77.4 ± 2.1	80.7 ± 0.9	>78 *	>83 *	59.9 ± 1.1
Gender F (%)	33.3	41.8	45	59.8	69.6
APOE4 (%)	30	58.2	13.5	52.4	38.4

Abbreviations: AD—Alzheimer’s disease; CTL—controls; CU—cognitively unaffected; DBCBB—Douglas-Bell Canada Brain Bank; F—female; PREVENT-AD—The PRe-symptomatic EValuation of Experimental or Novel Treatments for Alzheimer’s Disease. * The exact age at death is not provided when over 90 years old.

**Table 2:** *VAT1L* and *SERPINE1* polymorphisms.

SNP	Cohort	Freq CTL	Freq AD	CTL vs. AD *p* Value	SERPINE1 QTL *p* Value	Aβ42 CSF	pTau181 CSF	tTau CSF
rs17705051 (*VAT1L*)	PREVENT-AD	0.05	-	-	*p* = 5 × 10^−8^ (CSF)	*p* = 0.421	*p* = 6 × 10^−3^	*p* = 5 × 10^−3^
	ADNI	0.04	0.04	*p* = 0.801	*p* = 0.654 (CSF)	*p* = 0.642	*p* = 0.663	*p* = 0.629
	DBCBB	0.08	0.08	*p* = 0.972	*p* = 0.420 (Brain)			
	MAYO	0.04	0.05	*p* = 0.703	*p* = 0.413 (Brain)			
rs6092 (*SERPINE1*)	PREVENT-AD	0.12	-	-	*p* = 2.5 × 10^−2^ (CSF)	*p* = 0.067	*p* = 0.674	*p* = 0.870
	ADNI	0.09	0.12	*p* = 0.537	*p* = 7 × 10^−4^ (CSF)	*p* = 0.322	*p* = 0.461	*p* = 0.381
	DBCBB	0.13	0.22	*p* = 0.184	*p* = 0.792 (Brain)			
	MAYO	0.14	0.11	*p* = 0.311	*p = 4.1* × *10^−2^* (Brain)			

Abbreviations: AD—Alzheimer’s disease; ADNI—Alzheimer’s Disease Neuroimaging Initiative; CTL—control; DBCBB—Douglas-Bell Canada Brain Bank; Freq—frequency; PREVENT-AD—the PRe-symptomatic EValuation of Experimental or Novel Treatments for Alzheimer’s Disease; SNP—single nucleotide polymorphism.

## Data Availability

Data collected from the Douglas-Bell Canada Brain Bank are not publicly available; however, data can be provided by the corresponding author upon reasonable request. Gene expression data from the Mayo Clinic were downloaded from AMP-AD Knowledge Portal https://adknowledgeportal.synapse.org (synapse ID: syn5550404, accessed on 1 November 2024). CSF, genetic and clinical data from the ADNI cohort were downloaded from the ADNI website (http://adni.loni.usc.edu/, accessed on 1 November 2024) following registration. The PREVENT-AD multimodal data, including subject characteristics, CSF biomarkers levels, tau and amyloid-PET SUVR, APOE genotypes and RBANS scores, are summarized in the supporting data files from the PREVENT-AD data release 7, available from the corresponding authors upon reasonable request at https://openpreventad.loris.ca/ (accessed on 1 November 2024) and following registration.

## Supplementary Materials

The following supporting information can be downloaded at: https://www.mdpi.com/article/10.3390/genes16070818/s1. Figure S1: CSF SERPINE1 levels according to diagnostics in the ADNI cohort; Figure S2: CSF SERPINE1 levels compared to AD biomarkers in the ADNI cohort; Figure S3: CSF SERPINE1 levels compared to hippocampus volume in the ADNI cohort; Figure S4: Genome-wide association with CSF SERPINE1 protein levels in the PREVENT-AD cohort; Figure S5: Locus-wide association with CSF SERPINE1 protein levels in the ADNI cohort; Figure S6: SERPINE1 string network.

## Abbreviations

The following abbreviations are used in this manuscript:

CSF	Cerebrospinal fluid
PAI	Plasminogen activator inhibitor
AD	Alzheimer’s disease
PREVENT-AD	The PRe-symptomatic EValuation of Experimental or Novel Treatments for Alzheimer’s Disease
ADNI	The Alzheimer’s Disease Neuroimaging Initiative
DBCBB	Douglas-Bell Canada Brain Bank
tPA	Tissue plasminogen activator
uPA	Urokinase-type plasminogen activator
Aβ	Amyloid beta
MCI	Mild cognitive impairment
PSP	Progressive supranuclear palsy
CU	Cognitively unimpaired
GAP43	Growth-associated protein 43
SNAP25	Synaptosome-associated protein 25
SYT1	Synaptotagmin 1
APOE	Apolipoprotein E
CTL	Controls
NFT	Neurofibrillary tangles
pQTL	Protein quantitative trait loci
VAT1L	Vesicle amine transport 1 like
GWAS	Genome-wide association study
PLAT	Plasminogen activator, tissue type
TP53	Tumour protein p53
IL6	Interleukin 6
GSK3B	Glycogen synthase kinase 3 beta
