# Supplementary material for: Brain and CSF Alzheimer’s Biomarkers Are Associated with SERPINE1 Gene Expression
Source: Genes (Basel). 2025 Jul 12;16(7):818. doi: 10.3390/genes16070818 (PMC12294632; doi:10.3390/genes16070818)
Supplement: Supplementary file 1 [file genes-16-00818-s001.zip › genes-3733409-supplementary.pdf]

Workflow

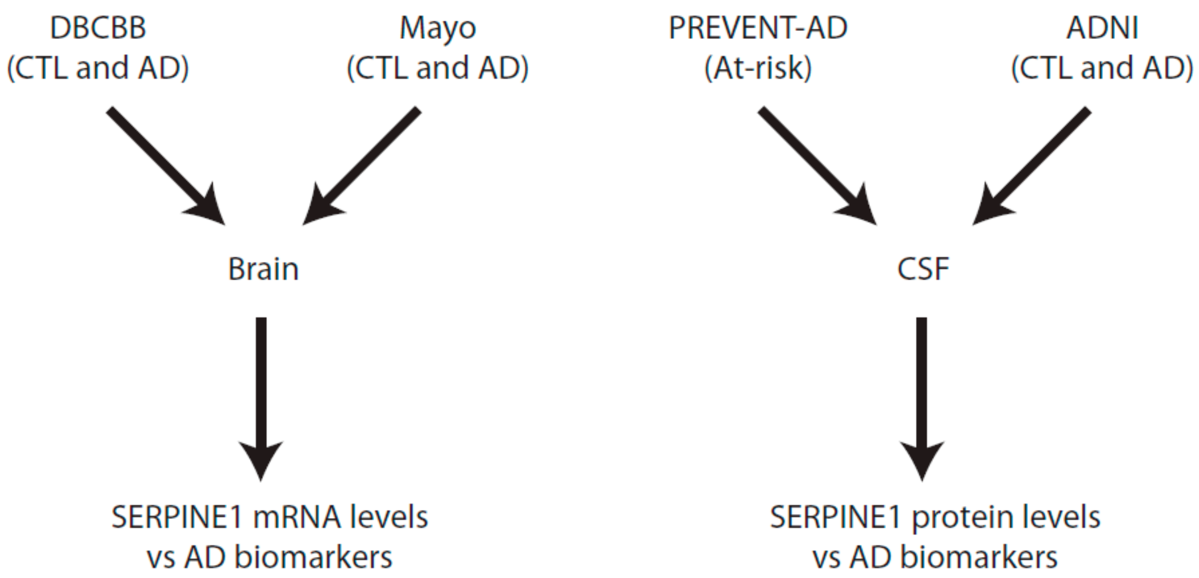

Replication using the Alzheimer's Disease Neuroimaging Initiative (ADNI) cohort

Data collection

Data used in the preparation of this article were obtained from the ADNI database (adni.loni.usc.edu). The ADNI was launched in 2003 as a public-private partnership, led by Principal Investigator Michael W. Weiner, MD. The primary goal of ADNI has been to test whether serial magnetic resonance imaging (MRI), positron emission tomography (PET), other biological markers, and clinical and neuropsychological assessments can be combined to measure the progression of mild cognitive impairment and early AD. For up-to-date information and details about MRI acquisition, see adni.loni.usc.edu.

CSF measurements

CSF Aβ42, pTau and tTau were standardized using the highly validated Roche Elecsys cobas e 601 fully automated immunoassay platform. CSF SERPINE1 levels were measured as part of a 190 analyte smultiplex immunoassay panel. The panel, referred to as the human discovery map, was developed on the Luminex xMAP platform by Rules-Based Medicine (RBM) to contain proteins previously reported in the literature to be altered as a result of cancer, cardiovascular disease, metabolic disorders and inflammation.

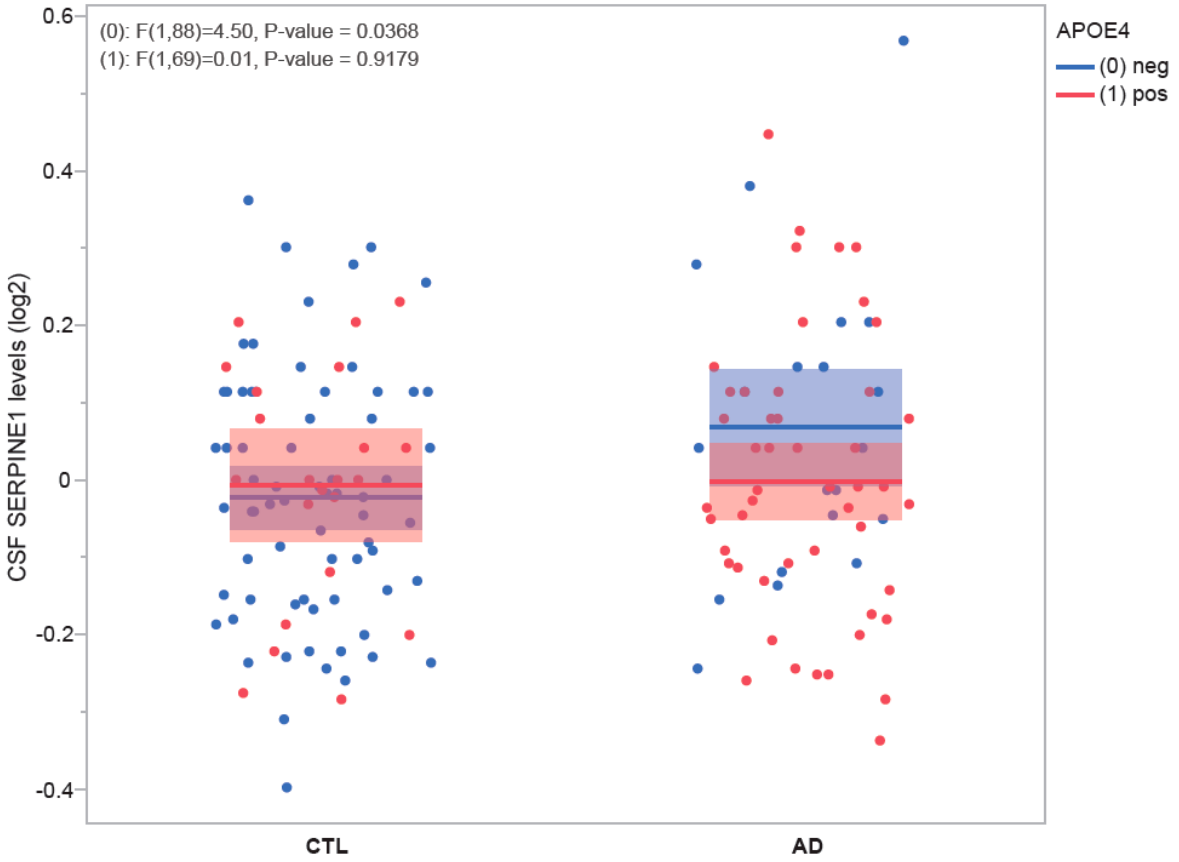

Figure S1. CSF SERPINE1 levels according to diagnostic in the ADNI cohort. In APOE4 negative subjects, a significant upregulation of SERPINE1 was observed when comparing AD patients to CTLs (p=0.04).

A)

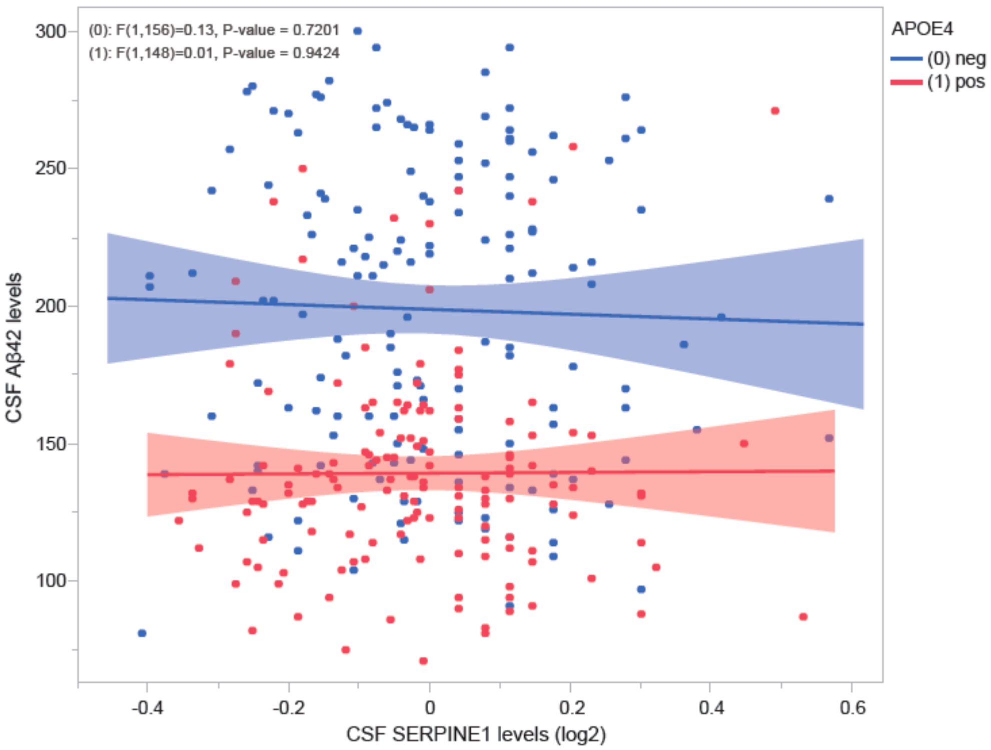
(0): F(1,156)=0.13, P-value = 0.7201
(1): F(1,148)=0.01, P-value = 0.9424
APOE4
(0) neg
(1) pos
CSF Aβ42 levels
CSF SERPINE1 levels (log2)
300
250
200
150
100
-0.4
-0.2
0
0.2
0.4
0.6

B)

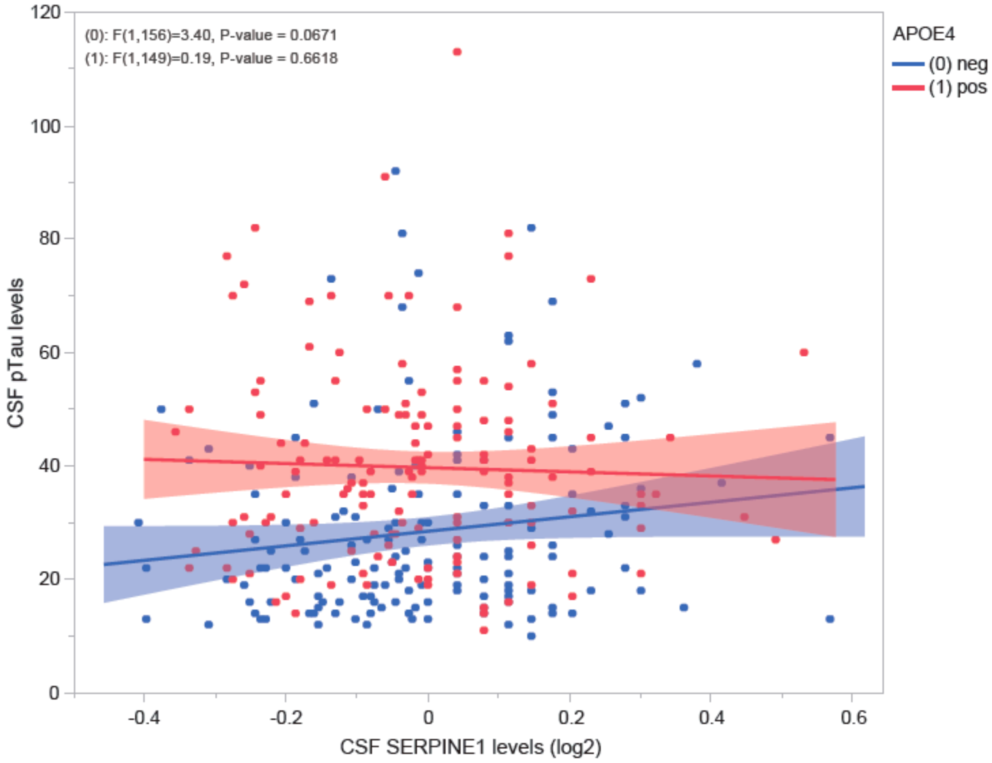
(0): F(1,156)=3.40, P-value = 0.0671
(1): F(1,149)=0.19, P-value = 0.6618
APOE4
(0) neg
(1) pos
CSF pTau levels
CSF SERPINE1 levels (log2)
120
100
80
60
40
20
0
-0.4
-0.2
0
0.2
0.4
0.6

C)

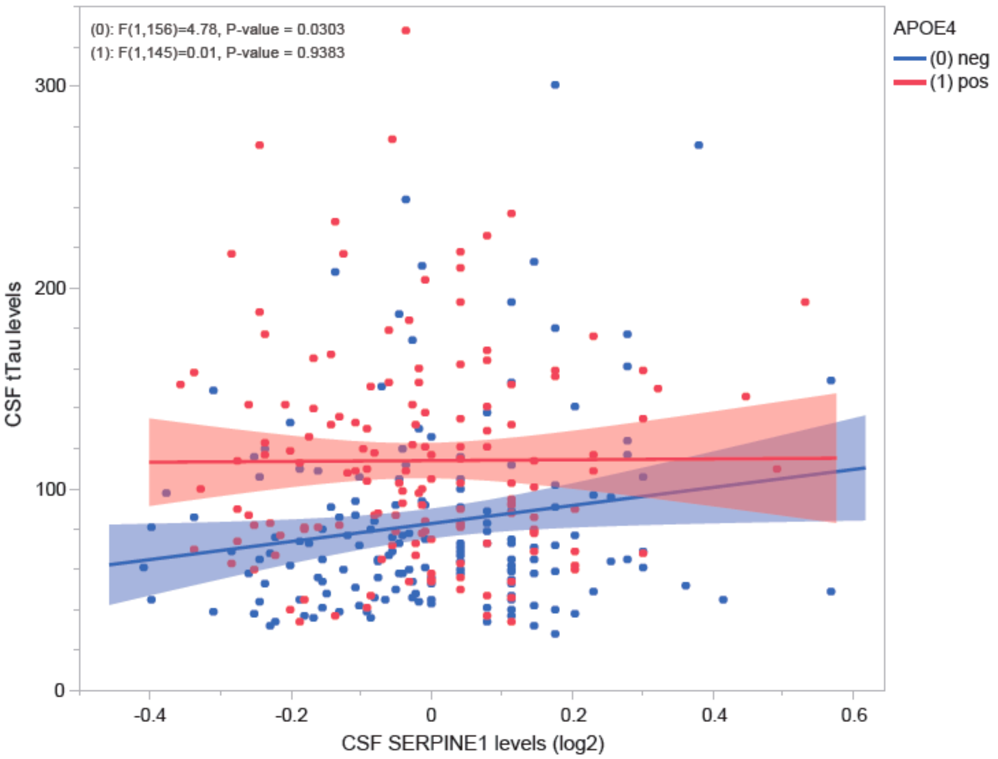

Figure S2. CSF SERPINE1 levels compared to AD biomarkers in the ADNI cohort. A) CSF Aβ42 levels did not correlate with SERPINE1 even after stratification for APOE4. B) In APOE4 negative subject, a correlation at a trend level was observed between CSF pTau levels and SERPINE1 (p=0.07). C) In APOE4 negative subjects, a significant correlation was found between CSF tTau levels and SERPINE1 (p=0.03).

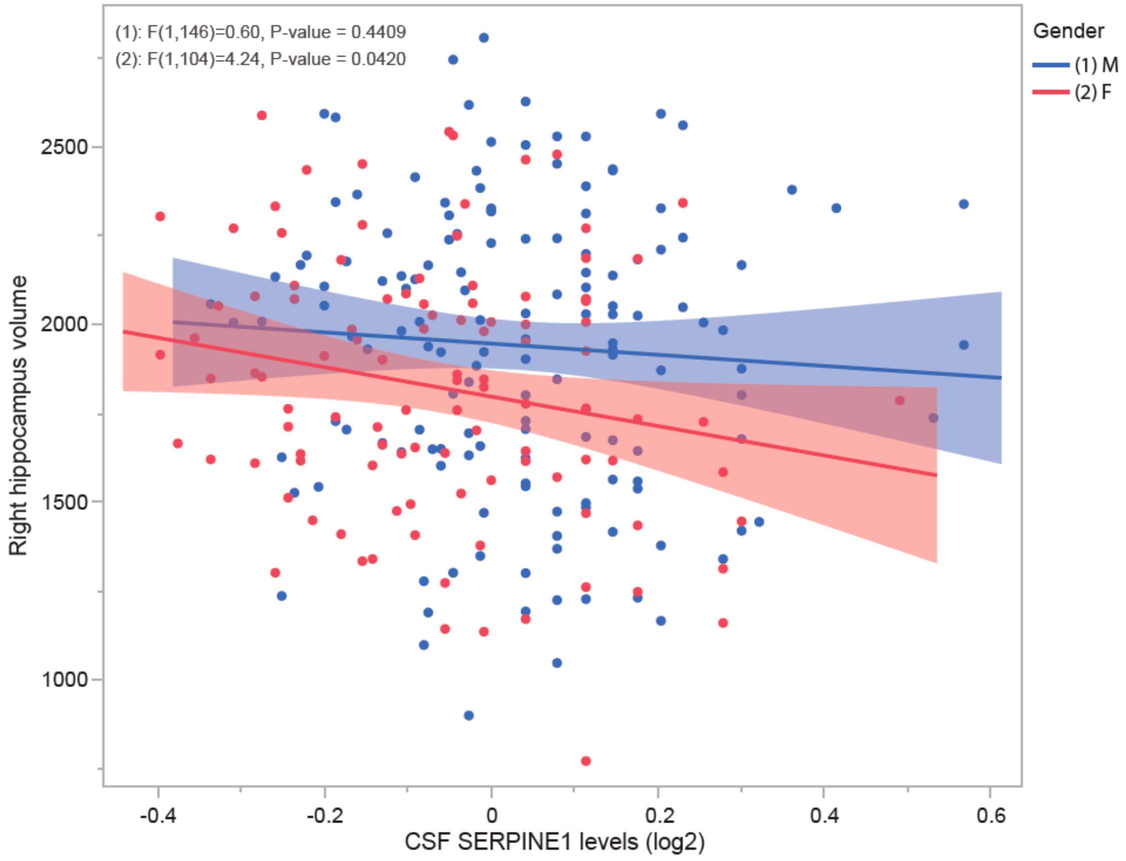

Figure S3. CSF SERPINE1 levels compared to hippocampus volume in the ADNI cohort. In females only, an inverse correlation was found between right hippocampus volume and CSF SERPINE1 levels (p=0.04). Note that no significant correlation was found between left hippocampus volume and CSF SERPINE1 levels (data not shown).

pQTL analysis with CSF SERPINE1 levels

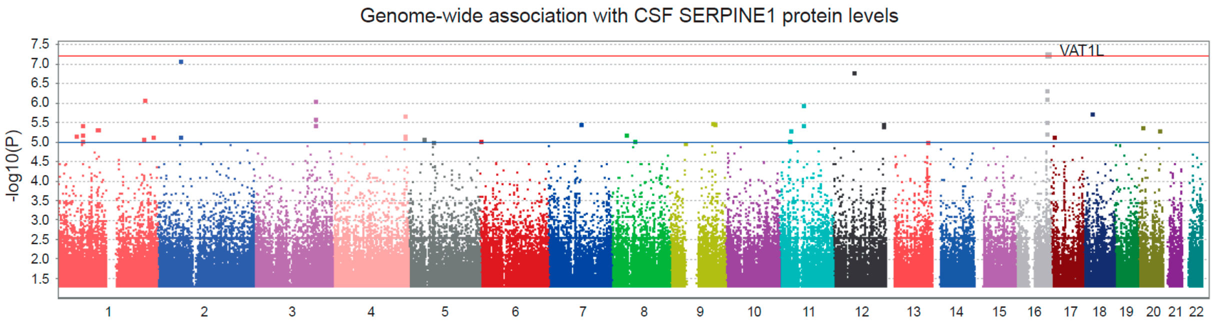

Figure S4. Genome-wide association with CSF SERPINE1 protein levels in the PREVENT-AD cohort. CSF SERPINE1 protein levels were measured with the help of OLINK technology in 87 asymptomatic individuals. 10 million imputed SNPs spanning the whole genome were contrasted with CSF SERPINE1 protein levels. The genome-wide significant threshold was set at a pvalue of $5x10^{-8}$ (-log10(P) of 7.3). A single SNP (rs17705051), found on chromosome 16 downstream of VAT1L, reached that threshold.

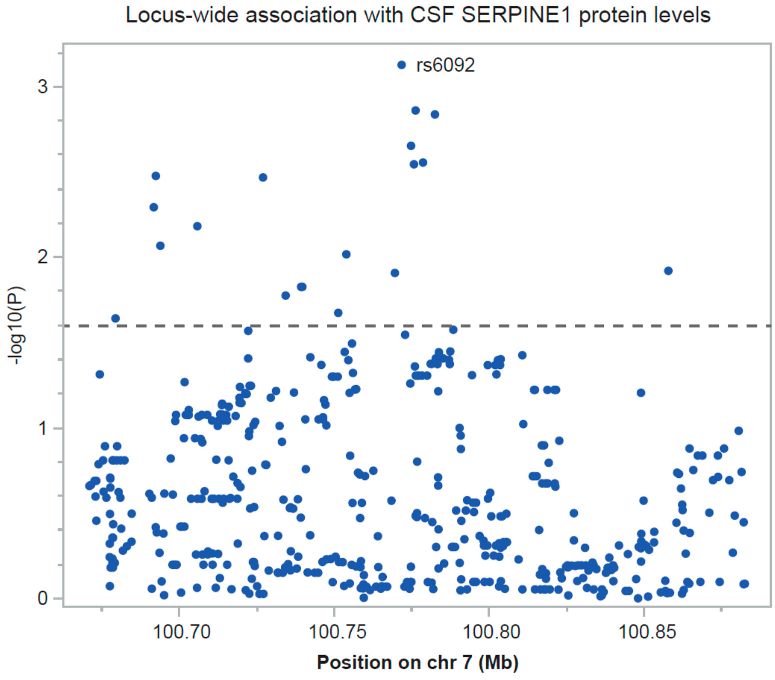

Figure S5. Locus-wide association with CSF SERPINE1 protein levels in the ADNI cohort. CSF SERPINE1 protein levels were measured with the help of SomaScan technology in 378 participants with or without cognitive decline. 515 SNPs were genotyped near SERPINE1 locus (+/- 100 Kb) using whole genome sequencing. Although not reaching genome-wide significance, rs6092 had the strongest association (p=0.0007). Dotted line represents a pvalue of 0.05 (-log10(P) of 1.3).

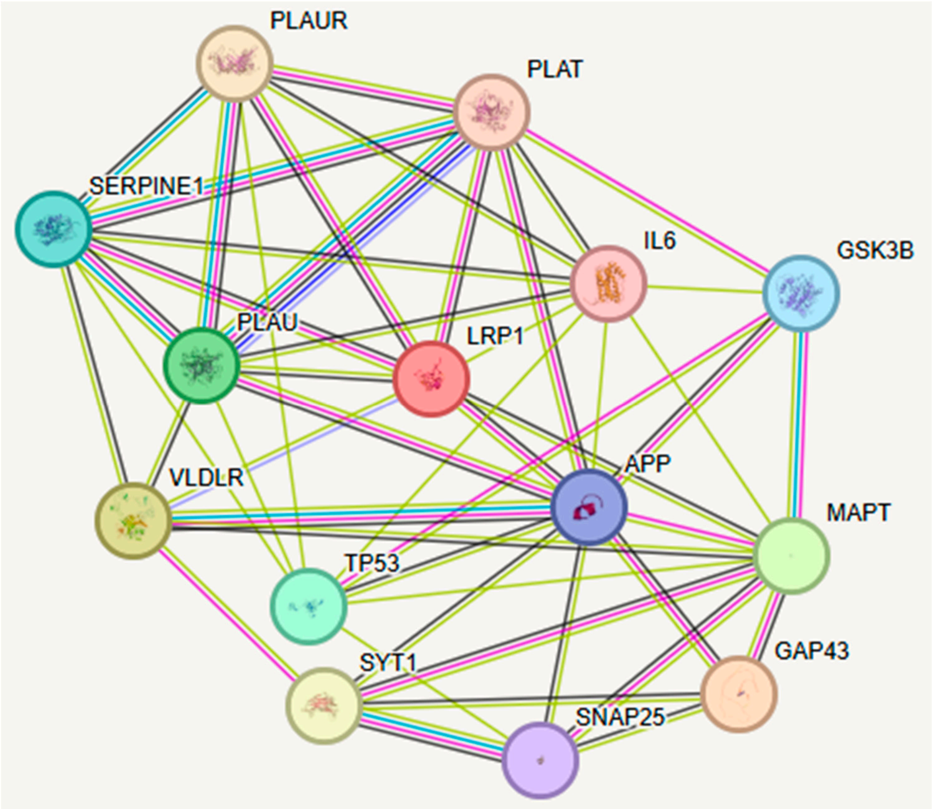

Figure S6. SERPINE1 string network. List of the main proteins involved in SERPINE1 pathway and their direct or indirect effect on amyloid (APP), tau phosphorylation (GSK3B -> MAPT) and the synaptic markers GAP43, SNAP25 and SYT1. Source: https://string-db.org.
